# Mechanisms of Allergen Immunotherapy in Allergic Rhinitis

**DOI:** 10.1007/s11882-020-00977-7

**Published:** 2020-12-12

**Authors:** Gabija Drazdauskaitė, Janice A. Layhadi, Mohamed H. Shamji

**Affiliations:** Immunomodulation and Tolerance Group, Allergy & Clinical Immunology, Inflammation, Repair and Development, National Heart & Lung Institute, Imperial College London, 1st Floor, Room 111, Sir Alexander Fleming Building, South Kensington Campus, London, SW7 2AZ UK

**Keywords:** Allergen immunotherapy, Allergic rhinitis, Innate and adaptive immune response, T cells, B cell, Innate lymphoid cells, Dendritic cells

## Abstract

**Purpose of Review:**

Allergic rhinitis (AR) is a chronic inflammatory immunoglobulin (Ig) E-mediated disease of the nasal mucosa that can be triggered by the inhalation of seasonal or perennial allergens. Typical symptoms include sneezing, rhinorrhea, nasal itching, nasal congestion and symptoms of allergic conjunctivitis. AR affects a quarter of the population in the United States of America and Europe.

**Recent Findings:**

AR has been shown to reduce work productivity in 36–59% of the patients with 20% reporting deteriorated job attendance. Moreover, 42% of children with AR report reduced at-school productivity and lower grades. Most importantly, AR impacts the patient’s quality of life, due to sleep deprivation. However, a proportion of patients fails to respond to conventional medication and opts for the allergen immunotherapy (AIT), which currently is the only disease-modifying therapeutic option. AIT can be administered by either subcutaneous (SCIT) or sublingual (SLIT) route. Both routes of administration are safe, effective, and can lead to tolerance lasting years after treatment cessation. Both innate and adaptive immune responses that contribute to allergic inflammation are suppressed by AIT. Innate responses are ameliorated by reducing local mast cell, basophil, eosinophil, and circulating group 2 innate lymphoid cell frequencies which is accompanied by decreased basophil sensitivity. Induction of allergen-specific blocking antibodies, immunosuppressive cytokines, and regulatory T and B cell phenotypes are key pro-tolerogenic adaptive immune responses.

**Conclusion:**

A comprehensive understanding of these mechanisms is necessary for optimal selection of AIT-responsive patients and monitoring treatment efficacy. Moreover, it could inspire novel and more efficient AIT approaches.

## Introduction

Allergic rhinitis (AR) is a chronic inflammatory disease of the lining of nasal mucosa induced by type I hypersensitivity response upon exposure to common inhaled allergens in sensitized individuals [1]. AR affects up to 40% of worldwide population with an increasing prevalence over the past 20 years [2–8]. It is associated with significantly lower quality of life due to impaired sleep, learning difficulties, deterioration of at-work performance and social functioning, which highlights AR as a substantial economic burden and a serious global health problem [8–11].

AR manifests itself through rhinorrhea, sneezing, nasal itching and congestion. Seasonal AR (SAR) is periodically triggered by outdoor allergens, in particular grass, tree or weed pollen. Symptoms of perennial AR (PAR) last throughout the year in response to persistently present indoor allergens, such as house dust mite (HDM), animal dander, insects and mold [12, 13]. It is noteworthy that a proportion of patients displaying nasal reactivity do not exhibit systemic sensitization evidenced by negative skin prick tests (SPT) and undetectable serum-specific IgE (sIgE), an endotype consequently defined as local AR (LAR) [14, 15]. Recent evidence shows that both sensitization patterns in response to different allergens can coexist within the same individual, the proposed term for which is dual AR (DAR) [16].

Currently employed pharmacotherapy approaches include antihistamines and intranasal corticosteroids which only provide a temporary symptomatic relief [12, 13]. Furthermore, such treatments fail to attenuate symptoms in 30% to 60% of the patients [17, 18]. Allergen immunotherapy (AIT) is a proposed treatment strategy for such individuals being the single disease-modifying strategy to date. Subcutaneous immunotherapy (SCIT) involves administration of incremental doses of sensitizing allergen for 8–12 weeks followed by high dose monthly interval over the course of 3–5 years. Sublingual immunotherapy (SLIT) involves administration of high doses of the allergen under the tongue. SCIT and SLIT confer long-term clinical benefit and immunologic tolerance after cessation treatment. While SCIT is highly efficient at inducing tolerance to both seasonal and perennial allergens, SLIT is regarded as a safer and more convenient alternative generally in managing SAR [19–21].

This review will focus on historical and recent advances in understanding the mechanisms of allergy and AIT in the context of AR. Furthermore, novel AIT approaches and predictive/ indicative biomarkers of treatment success will be discussed. Collectively, these findings can support new potential treatment approaches.

## Mechanisms of Allergic Rhinitis

### Sensitization

The primary step in the cascade of allergic inflammation is orchestrated by intricate interactions between epithelial (EC) and dendritic cells (DCs) which ultimately lead to the initiation of early and late phase responses. Initially, an inhaled allergen passes through ECs of the nasal mucosa. Once activated, these cells shed a range of chemokines, particularly CCL20, in an ADAM10-mediated manner, which in turn promotes the recruitment of immature DCs [22, 23].

A dysfunctional epithelial barrier can contribute to the pathophysiology of AR. Upregulated activity of histone deacetylase (HDAC) compromises epithelial integrity by impairing tight junction proteins, possibly escalating allergen challenge [24•]. Furthermore, necroptosis-induced release of nuclear IL-33 as well as secretion of TSLP and IL-25 from ECs introduce stimuli required for the development of a pro-allergic dendritic cell of type 2 (DC2) phenotype, defined by the expression of CD141, GATA-3, OX40L, and RIPK4 [25, 26]. A recent study highlighted that epithelium-derived cytokines exert their pro-inflammatory effects in the most severe manner when in combination, suggesting their functions are additive [27]. In addition, these mediators facilitate the development of group 2 innate lymphoid cells (ILC2s), which, together with DC2s, amplify local T helper type 2 (T_H_2)-mediated allergic inflammation [26, 28]. TSLP and IL-33 can also directly activate T_H_2 cells, as seen in a murine model of AR [29]. Recently, IL-33 was also established as a key facilitator of mast cell degranulation, resulting from the inhibition of ST2/PI3K/mTOR-mediated autophagy, thereby amplifying early phase responses [30].

After aeroallergen internalization, activated DCs migrate to local lymph nodes. There, major histocompatibility complex (MHC) II-dependent antigen presentation and CD80/CD86-mediated co-stimulation prompt naïve CD4^+^ T lymphocyte polarization into effector cells [31]. In atopic subjects, presence of IL-4 is key for the development of the T_H_2 subset [32]. Healthy subjects can also develop an allergen-specific T cell response, though contrastingly, interferon-γ (IFN-γ)-secreting T helper type 1 (T_H_1) polarization is favored [33]. Furthermore, non-coding RNA GAS5, secreted by the exosomes of the nasal epithelium in AR patients, has been found to downregulate expression of T_H_1-related transcription factor T bet henceforth suppressing T_H_1 differentiation [34•].

T follicular helper (T_FH_) cells are another key subset to arise in the germinal centers (GCs) of lymph nodes in response to DC-mediated antigen presentation. These CD4^+^ lymphocytes express a surface marker CXCR5 and a transcription factor Bcl6. CXCR5, mutually expressed by B lymphocytes, is crucial for B follicle formation and T and B cell interaction [35]. Besides the lymph nodes, effector T_FH_ cells can also enter the circulation or migrate to the nasal mucosa, where they can acquire T_H_2-like characteristics and induce local IgE production. Type 2 follicular helper T cells (T_FH_2) express transcription factor GATA-3 and secrete a T_H_2 cytokine repertoire [36, 37]. A recent study in mice by Gowthaman et al. showed that IL-13-secreting T_FH_ cells are required to facilitate affinity maturation and differentiation of IgE^+^ B cells [38]. Moreover, AR patients with or without asthma show significant elevation in circulating T_FH_2 numbers [39].

T_FH_-secreted IL-4, IL-13 and IL-21 together with T_H_2-derived IL-4 and IL-13 promote B cell ε-germline transcription. Class-switch recombination and B cell activation is finalized by CD40/CD40L interaction between T_FH_ and B cells [40]. The phenomenon of sensitization to allergen occurs when IgE^+^ plasma cells produce sIgE which binds to high affinity FcεRI receptors on the surface of mast cells and basophils. Upon subsequent exposure, the allergen cross-links neighboring IgE molecules and induces degranulation, leading to the early phase responses [38, 41].

### Early Phase Responses

**Fig. 1:**
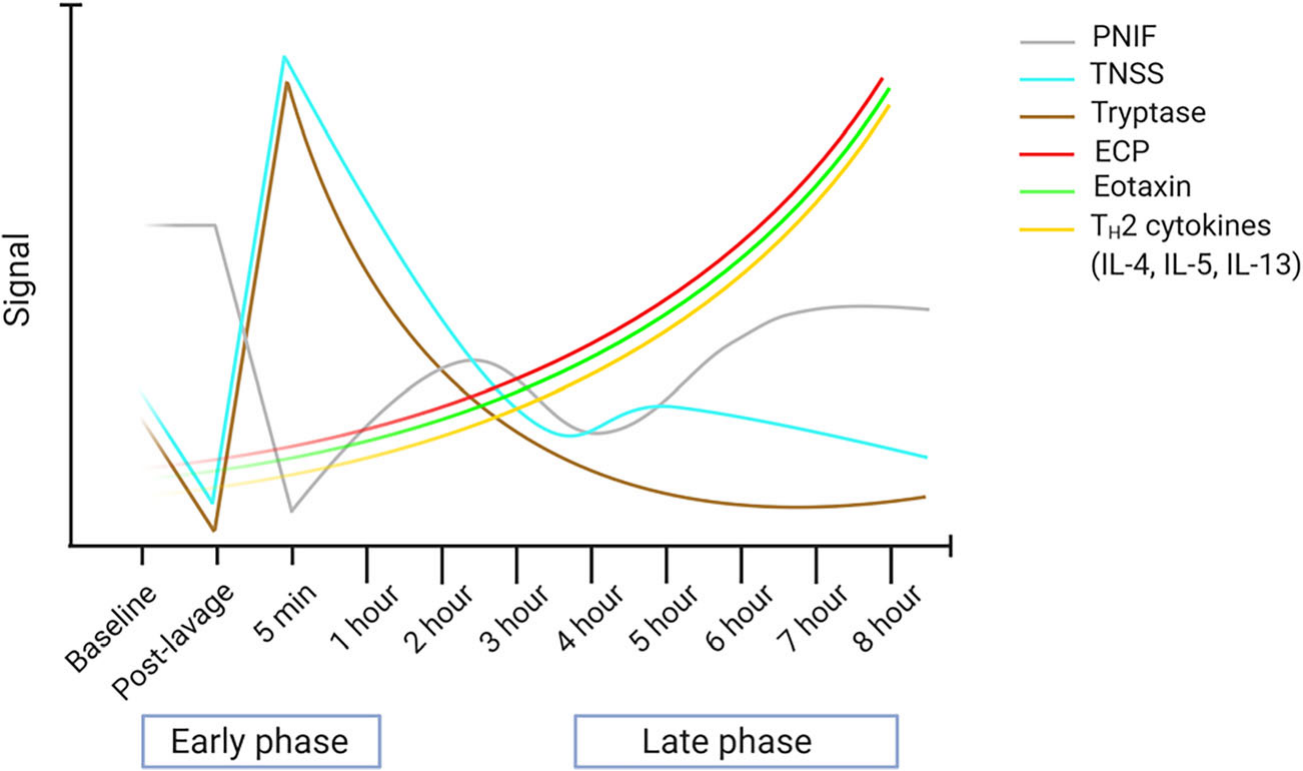
The biphasic response of allergic rhinitis following nasal allergen challenge (NAC). In the early phase, tryptase and total nasal symptom scores (TNSS) peak at 5 min post challenge which is accompanied by deterioration of peak nasal inspiratory flow (PNIF). Eosinophilic cationic protein (ECP), eotaxin, and T_H_2-related cytokines gradually increase and peak at 8 h during the late phase response which is paralleled by ongoing nasal congestion. Created with BioRender.com

The early phase responses (EPR) take place for up to 60 min post-nasal allergen challenge (NAC). During EPR, levels of tryptase in nasal fluid following NAC significantly peak at 5 min post exposure. This elevation is accompanied by severe rhinorrhea, sneezing, itching and nasal obstruction, evident by peaks in total nasal symptom score (TNSS). Moreover, a significant deterioration of peak nasal inspiratory flow (PNIF), a surrogate of nasal congestion, gradually recovers to baseline at 3–4 h post NAC (Fig. 1) [42].

A key mechanistic feature of the early phase reactions is IgE-dependent mast cell and basophil degranulation [43]. High affinity IgE receptors preferentially bind free IgE molecules, whereas IgE-allergen immune complexes are cleared upon binding to low-affinity receptor CD23 on B cells [44]. FcεRI are expressed not only on basophils and mast cells, but DCs as well. The expression of high-affinity receptors can be elevated in response to increasing serum IgE. Irrespective of allergic status, IgE primarily binds to basophil rather than DC receptors [45]. Just recently, it was demonstrated that upon allergen exposure, increases in serum IgE are accompanied by expansion of IgE^+^ plasmablasts. Moreover, IgE-related memory cells were found to reside in the allergen-specific IgG^+^ B cell fraction [46•].

Following the allergen cross-linking of adjacent IgE molecules, the mediator release from intracytoplasmic granules in the airway is orchestrated by phosphodiesterase 3 which mediates intracellular signaling of cGMP and cAMP [47]. The contents released include histamine, tryptase, cysteinyl leukotrienes and prostaglandin D2 [43, 47]. Notably, overexpression of CD300c, which acts in a co-stimulatory manner for IgE-dependent basophil activation, is seen in patients with AR [48].

Stimulation of histamine receptors H1 and H2 located on sensory neurons causes itching and sneezing, whereas stimulation of these receptors on ECs results in downregulation of tight junctions, thus increasing vascular permeability. Locally produced histamine in nasal secretions of AR patients is sufficient to compromise epithelial integrity in vitro, which is essentially a mechanism of continuous exacerbation of the allergic cascade [49]. This, together with cysteinyl leukotriene and prostaglandin D2-mediated chemoattraction, promotes immune cell influx to the nasal mucosa [50].

Although basophils and mast cells share numerous functional similarities, recent findings have elucidated regulatory differences of the effector functions. Several studies suggest that basophils and mast cells have different thresholds of stimulation to achieve FcεRI-dependent activation [51–53]. Moreover, studies show that mast cell survival is actively promoted by monomeric IgE binding to FcεRI which in turn induces an autocrine secretion of IL-3. While basophils similarly respond to IL-3, the induction of the cytokine is not subjected to monomeric IgE [54]. Besides promoting basophil survival, pre-exposure to IL-3 can also enhance histamine and pro-T_H_2 cytokine production upon IgE-allergen cross-linking [55]. Recent in vitro studies have demonstrated that peripheral basophils can be activated in an allergen-independent manner by cell-cell contact with ECs [56] or by high concentrations of serum IgE [57].

### Late Phase Responses

Late phase responses (LPR) occur at 4 to 12 h post allergen challenge and are generally characterized by tissue recruitment of eosinophil, T_H_2 cells, and ILC2s [43, 58]. Significant increases in nasal eotaxin, eosinophil cationic protein (ECP), and T_H_2-related cytokines IL-4, IL-5, IL-9, and IL-13 are detected within 8 h of NAC. Elevation of IL-5 and IL-13 inversely correlates with PNIF, making nasal obstruction the clinical hallmark of late phase reactions (Fig. 1) [42].

A wide repertoire of T_H_2-derived cytokines (IL-4, IL-5, IL-9, IL-13) orchestrates a variety of critical allergic reactions. Although T_H_2 phenotype develops in response to basophil-derived IL-4, subsequently differentiated T_H_2 cells secrete IL-4 in an autocrine manner to maintain their identity [32, 59]. A study in a mouse model of HDM-allergic airway inflammation showed that T_H_2-derived IL-4, similarl to histamine, can contribute to the disruption of mucosal barrier by tight junction downregulation [43, 49]. IL-4 and IL-13 upregulate endothelial adhesion molecules, such as ICAM-1 and VCAM-1, to facilitate migration of effector cells to the nasal mucosa [60]. IL-5 is critical for tissue eosinophilia, as it not only promotes their release from bone marrow but also inhibits their apoptosis [61, 62]. A similar cell survival-promoting effect of this cytokine was recently observed in CD4^+^ T cells of AR patients [63]. Locally recruited eosinophils secrete toxic mediators which damage the nasal epithelium [64, 65]. Finally, T_H_2-derived IL-9 promotes mast cell differentiation and maturation [66].

T_H_2 cells express CRTH2 which is a receptor for prostaglandin D2 secreted during the EPR. The expression of this receptor on T_H_2 cells is regulated by tyrosine kinase and was found to be elevated 6 h post NAC suggesting it is the peak of T_H_2 migration [67]. Furthermore, a study exploring the spectrum of AR has identified T_H_2 cells highly expressing ST2, a receptor for IL-33, as the most pathological subset, which is not present in asymptomatic sensitized patients. The evolvement of this phenotype could be the immunological shift required for the clinical manifestation of AR [68•]. A relatively novel subset of allergen-specific T_H_2 cells (T_H_2A), virtually absent in non-atopic subjects, was found to be key in promoting a swift response to an allergen [69•]. Furthermore, T_H_17-derived IL-17 was found to be elevated in the serum of AR patients. This cytokine can potentially contribute to the pathology of AR by promoting sIgE synthesis in B cells [70, 71].

Accumulating evidence suggests that type 2 inflammation is not the sole driver of the LPR. A study involving nasal mucosa of grass pollen (GP) allergic patients showed that late phase responses also involve upregulation of genes related to alternative complement pathway (factor P and C5AR1) and the inflammasome components (IL-1α and IL-1β), the latter being shown to promote neutrophil recruitment [72]. Moreover, a study on birch pollen-allergic peripheral blood neutrophils demonstrated their capability to fully process and present the allergen subsequently promoting Bet v 1-specific T cell proliferation and cytokine production [73•]. In a HDM-sensitized murine asthma model, inhibition of the complement component C5a was shown to reduce T_H_2 infiltration and IL-4 concentration in the lung tissue without affecting their counterparts ILC2s, suggesting it has a role in amplifying type 2 inflammation [74].

Recent studies in the context of AR have highlighted the emerging role of PD-1/PD-L1 axis among its immune checkpoint counterparts. Soluble PD-L1, significantly more abundant in the circulation of healthy subjects compared to AR, was shown to negatively correlate with IL-4 as opposed to being positively associated with IFN-γ which suggests that T cell exhaustion is a potential protective mechanism against the disorder [75]. The blockade of this co-inhibitory axis can indeed promote allergen-specific CD4^+^ T responses in cases of both PAR and SAR. However, given that it affects both T_H_1 and T_H_2 cells in vitro, the contributions of this mechanism could be studied further considering possible local or cell-cell interactions [76].

ILC2s are innate counterparts of T_H_2 cells. They share the expression of CRTH2 but are lineage negative and act in an antigen-independent manner [28]. It was shown that upon stimulation with epithelial cell-derived TSLP and IL-33, ILC2s actively reset their miRNA repertoire, similar to the behavior of T cells after antigen stimulation. miR-19a in particular is crucial for the regulation of IL-13 production [77]. A study by Miao et al. demonstrated an increase in circulating IL-13^+ ^ILC2s together with a greater capacity of this subset to produce IL-13 in response to IL-33 and IL-25 which was observed during natural pollen season in mug-worth sensitized asthmatics [78]. Similarly, SAR patients were shown to have elevated frequencies of total ILC2 and IL-13^+ ^ILC2 during grass pollen season, compared to out-of-season which correlated with seasonal symptom severity [79]. To support that, an in-season study involving grass pollen-sensitized patients of allergic rhinoconjunctivitis showed a significant increase in circulating ILC2 numbers. Here, ILC2s did not demonstrate an enhanced ability to produce IL-4 and IL-13 after in vitro stimulation with phorbol 12-myristate 13-acetate and ionomycin. In the allergic group, the functional impairment was rather observed in ILC1s, the counterparts of the T_H_1 subset, as evidenced by reduced IFN-γ production [80].

### Mechanisms of AIT in Allergic Rhinitis

**Fig. 2:**
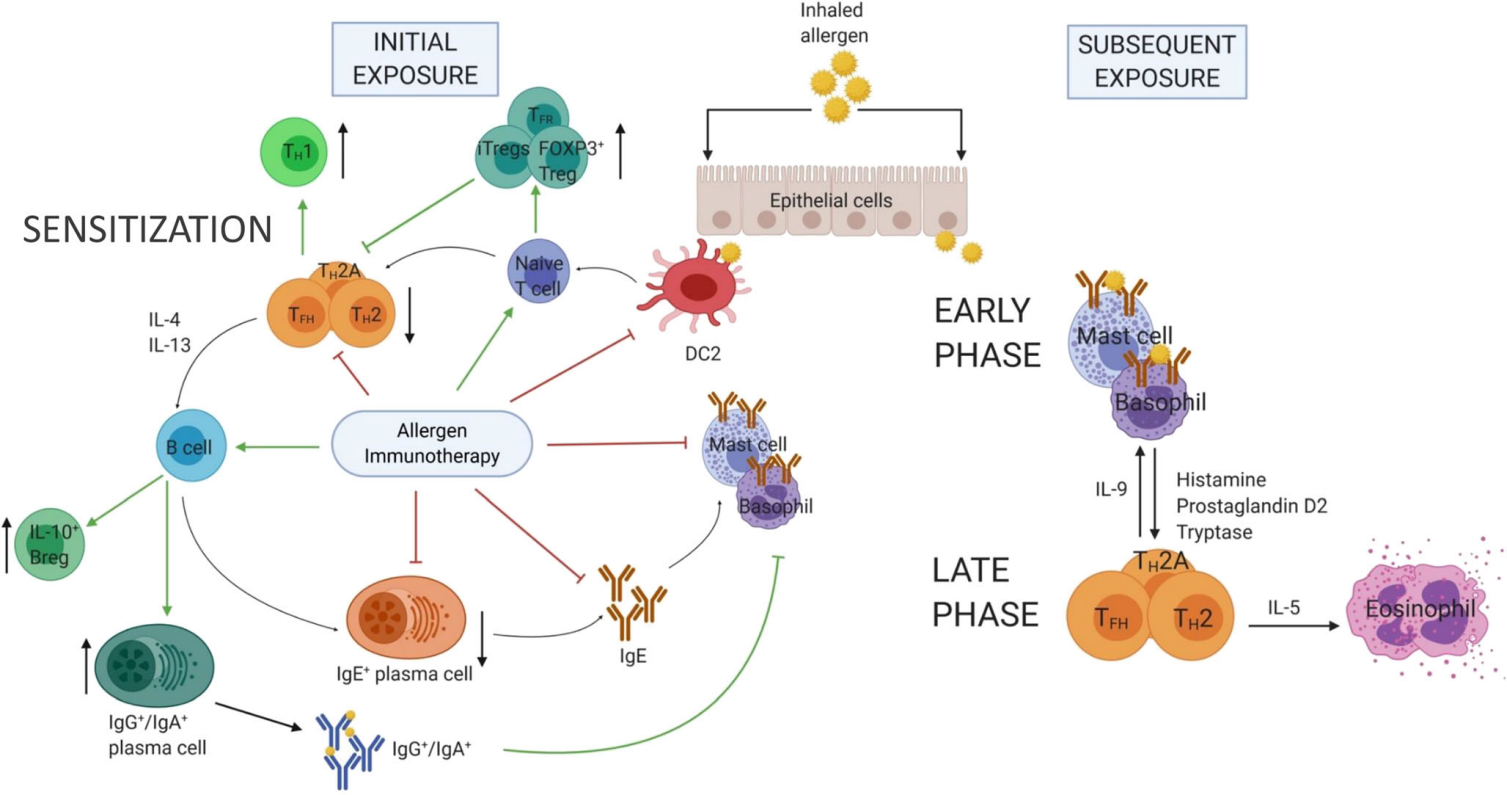
Mechanisms of allergic sensitization and allergen immunotherapy (AIT). (A) Upon inhalation of the allergen, ECs recruit DCs and polarize them to a pro-allergic DC2 phenotype. These cells uptake the allergen and migrate to lymph nodes, where they present it to naïve T cells and promote the development of T_H_2 and T_FH_ subsets. T_FH_ and T_H_2 cells collectively facilitate B cell maturation and class-switch recombination which leads to allergen-specific IgE production. These IgE molecules bind to high-affinity receptors on basophil and mast cell surfaces this way sensitizing the patient. The early phase reactions are triggered when a sensitized individual is subsequently exposed to the allergen, which in turn cross-links neighboring IgE molecules on basophil and mast cell surfaces and prompts the release of vasodilatory and chemoattractive mediators. This facilitates the recruitment of late phase effector T cells and eosinophils. (B) AIT suppresses the development of DC2 phenotype and promotes naïve T cell differentiation to regulatory phenotypes (iTregs, FOXP3^+^Tregs, T_FR_ cells). These subsets in turn suppress T_H_2, T_H_2A and T_FH_ responses and favor the differentiation of T_H_1. Inhibition of T_H_2 responses results in reduced local eosinophilia and prevents the development of IgE^+^ plasma cells. AIT also induces Bregs and IgG^+^/IgA^+^ plasma cells which produce blocking antibodies that compete with IgE for binding to the allergen, preventing the cross-linking of high-affinity receptors on mast cell and basophil surfaces and inhibiting their degranulation. Red arrows represent inhibition of effector cells; green arrows represent AIT-induced regulatory phenotypes and their effects; black arrows represent increases or decreases of the population frequencies. *EC*, epithelial cells; *DC*, dendritic cells; *T*_*H*_*2*, T helper type 2 cell; *T*_*FH*_, T follicular helper cell; *Treg*, T regulatory cell; *Breg*, B regulatory cell. Created with BioRender.com

AIT is a standard therapeutic approach that is indicated for those AR patients whose symptoms persist in spite of consumption of conventional anti-allergic medication. Numerous double-blind, placebo-controlled clinical studies have demonstrated that both SCIT and SLIT are effective options for managing seasonal and perennial allergies. Critically, on-going treatment-induced desensitization can translate into long-term allergen-specific tolerance and clinical benefit, lasting for 2 to 3 years after its cessation [19–21]. Accumulating evidence fuels a more comprehensive understanding of AIT-related mechanisms of tolerance (Fig. 2; Table 1).

**Table 1:** A summary of key cell subsets, their role in allergic rhinitis (AR), and allergen immunotherapy (AIT)-induced effects on their responses. *EC*, epithelial cells; *DC2*, dendritic cells of type 2; *ILC2s*, group 2 innate lymphoid cells; *T*_*H*_*2*, T helper type 2 cell; *T*_*FH*_*2*, type 2 follicular helper T cells

Stage of allergy	Cell subset	Key functional molecules	Role in allergic rhinitis	Effects of allergen immunotherapy
Sensitization	EC	CCL20 [23, 24•] TSLP, IL-25, IL-33 [26, 28] RNA GAS5 [35]	DC recruitment [24•] Induction of ILC2 [29] and DC2 [27] cell subsets Promotion of T_H_2 [30] and inhibition of T_H_1 [35] polarization Promotion of mast cell degranulation [31]	Restoration of EC integrity in murine models [81]
	DC2	MHC II CD80/CD86 [32]	T_H_2 polarization Antigen presentation [32]	Promotion of DCreg polarization [27, 82] Increased FcεRI expression [46•]
	T_FH_2	Surface CD40 secreted IL-4, IL-13, IL-21 [36, 39]	Induction of B cell ε-germline transcription, differentiation and affinity maturation and sIgE production [39, 41]	Decreased circulating numbers [38, 83] Restored function of suppressive counterparts T_FR_ cells [84]
	B cells	IgE [42]	Mast cell and basophil sensitization [42] Antigen presentation [85••]	Induction of IgG^+^ B cells [39, 47, 86–88•] Induction of IL-10^+^Bregs [87–90] Reduced CD23 expression [85••]
Early phase responses	Basophils	Histamine, tryptase, cysteinyl leukotrienes, PGD2 [43, 44, 50, 51]	Promotion of T_H_2 polarization [33] Activation of ILC2s [51] Increasing vascular permeability and immune cell influx [50] Induction of symptoms: itching, sneezing, rhinorrhea [44]	Reduced infiltration [91–94] Decreased sensitivity and degranulation [95, 96••, 97, 98]
	Mast cells			
Late phase responses	T_H_2	IL-4, IL-5, IL-9, IL-13 [44, 50, 64, 67, 99]	Induction of B cell ε-germline transcription, differentiation and affinity maturation [41] Downregulation of tight junctions of nasal epithelium [50] Upregulation of endothelial adhesion molecules [61] Promotion of basophil and mast cell differentiation [67] Eosinophil recruitment and survival [62, 63]	Induction of iTregs [100–103] and nTregs [103–106] Reduced T_H_2 and increased T_H_1 frequencies and corresponding cytokines [100, 107–112]
	ILC2s			Reduced circulating frequencies [80, 113•]
	Eosinophils	LTC4, PGE2, EDN, ECP [66]	Damage of the nasal epithelium [65]	Reduced infiltration [92, 104, 107, 114, 115]
	Neutrophils	MHC II [74]	Antigen presentation [74]	Reduced activation (157)

### Effect of AIT on Innate Immune Responses

Innate responses are triggered by initial allergen interactions with the physical barrier—the nasal epithelium. To date, no studies in humans have demonstrated a reduction of epithelium-derived pro-inflammatory cytokines following AIT. However, a recent study of SCIT in a murine Der f-sensitized model demonstrated a reduced IL-25 secretion and restoration of epithelial integrity by ameliorating said cytokine-related endoplasmic reticulum stress and EC apoptosis [99]. However, such findings remain to be replicated in human models. The subsequent allergen presentation by DCs bridges the innate and adaptive immunity. Historically, AIT-induced elevation of complement component 1Q (C1Q)-expressing DCreg phenotype has been associated with the response to treatment. This DC subset favors the development of regulatory T cell phenotype [26, 81]. More recently, HDM-SCIT was shown to induce a temporary increase in FcεRI expression on DCs, suggesting that IgE/FcεRI signaling in this subset can contribute to the development of tolerance [45].

The EPR are mediated by mast cells and basophils which act in an antigen-independent manner. AIT reduces the infiltration of these effector cells which is followed by decreases of histamine and tryptase in the nasal mucosa of AR patients [82, 91–93]. A recent study involving SCIT for GP allergy demonstrated a 447-fold decrease in basophil sensitivity to the allergen 1 year into the treatment. The trend remained similar a year following treatment cessation. Remarkably, long-term clinical efficacy correlated with the reduction of basophil sensitivity 3 weeks into the treatment suggesting this could be a potential predictive biomarker [94]. A strong correlation between CD203c expression and clinical efficacy of SLIT for *Parietaria* was documented in a randomized 12-month trial. Interestingly, the reference group treated with conventional medication exhibited slightly reduced threshold of basophil activation, which highlights that AIT can serve to not only treat the disease but also prevent its progression [95]. Recently, an alternative to surface marker expression for evaluating basophil function has been described. Principally, the histamine amount released from the cell inversely correlates with intracellular fluorochrome-labeled diamine oxidase (DAO). Upon ex vivo basophil stimulation with GP allergen, the frequency of DAO^+^ basophils was significantly higher in SCIT and SLIT-treated compared to the untreated group. Critically, this suppression of histamine release correlated with the alleviation of clinical symptoms [96••]. A proteomics approach has been implemented in a study investigating cedar pollen (CP)-SLIT effects on mast cell degranulation, as responder and non-responder groups were not distinguishable by serum IgE, IgG or relevant cytokines. Thrombospondin 1 (THRS-1) was identified as a significant suppressant of mast cell degranulation elevated in the responder group compared to non-responders [97].

In the following LPR, the main innate cell populations are eosinophils and ILCs. Reduced local eosinophil infiltration is a characteristic feature resulting from AIT-induced dampening of upstream T_H_2 responses together with reduced eotaxin concentrations in nasal fluids [91, 98, 104, 114, 115]. ILCs are the sole innate immunity component of the lymphoid lineage. A murine airway inflammation model demonstrated a reduced proportion of IL5^+ ^ILC2s in the circulation without affecting innate ILC-activating cytokines IL-33 and IL-25 after a 2-month birch pollen-SCIT [107]. In contrast, a 4-month GP-SCIT in humans failed to induce alterations of ILC2 frequencies in the periphery [80]. The first evidence of the effects of AIT on circulating ILC2s in humans was reported by Lao-Araya et al. Here, GP-SCIT for 8 months and more reduced circulating total and IL-13^+^ ILC2 frequencies in SAR patients during the pollen season which was accompanied by reduced seasonal symptoms [79]. In the context or PAR, responders to a 2-year HDM-SCIT exhibited a significant decrease in circulating ILC2 and elevation of ILC1 frequencies compared to non-responders and the untreated group, ultimately achieving a similar ILC2/ILC1 proportion as seen in healthy subjects. Moreover, ex vivo ILC2 stimulation with Der p1, IL-33 and IL-2 showed that ILC2s in AIT-treated group exhibit a reduced expression of activation marker CD69, however, without any impairment of cytokine secretion [116]. Very recently, a novel regulatory ILC (ILCreg) subset has been described. ILCregs were demonstrated to suppress lung [113•] and intestinal [117] inflammation by secreting IL-10. Following this, Morita et al. were able to demonstrate an in vitro induction of ILCreg subset derived from ILC2s upon exposure to retinoic acid (RA) and the presence of IL-2 and IL-33. ILCregs demonstrated a dose-dependent IL-10 production in response to RA, while IL-5 and IL-13 were not elevated. Transcriptome profiling of sorted IL-10^+^ ILCs revealed a downregulation of ILC2-related genes, such as CRTH2 and CD127 with conversely elevated Treg-related CD25 and cytotoxic T lymphocyte-associated protein 4 (CTLA-4) expression. However, IL-10 production rather than CTLA-4 activity was critical for ILCreg-dependent anti-proliferative activity toward CD4^+^ T cells and ILC2s. Concordantly, in human nasal tissue, epithelial cell-derived RA was also shown to promote ILCreg development from ILC2s. Conclusively, this novel subset dampens excessive inflammation [118]. The potential role of ILCregs in AIT-induced tolerance remains to be investigated in future studies.

## Effect of AIT on Adaptive Immune Responses

### Effect of AIT on T_H_2 and T_FH_ Cells

Given the pivotal role of the T_H_2 subset in mediating allergic inflammation, dampening their response is a preferred outcome of AIT. Typically, immune deviation toward T_H_1 polarization is one of the mechanisms related to tolerance, particularly given that such response to an allergen is evident in healthy patients [33]. Generally, T_H_2 and T_H_2A numbers and their cytokines can be reduced systemically and locally following conventional AIT approaches [100, 108, 109, 119]. Complementary to this, AIT is associated with an increase in T_H_1 numbers, related chemokines and IFN-γ [109–111, 115].

SLIT for *Artemisia annua* pollen showed a reduction in T_H_2 proportion as early as 16 weeks which was accompanied by clinical symptomatic relief. Although the treatment course was comparably shorter than described in other studies, tolerance persisted throughout the following pollen season [108]. A recent study in timothy grass pollen-allergic children confirms that 3-year treatment can lead to a sustained clinical benefit. Two years after treatment discontinuation, systemic and local tolerance was paralleled by low ratios of IL-5/IFN-γ, and IL-13/IFN-γ. The study also detected elevated T_H_1-related chemokines CXCL10 and CXCL11, suggesting that sufficient migration of the subset to the site of inflammation can also be crucial for tolerance [111]. An indirect role for the regulation of T_H_1-favored immune deviation was attributed to type I IFN signaling. Gene expression analysis in SAR patients after 1 year of SLIT showed a significant downregulation of type I IFN signaling pathway-related genes (STAT1, STAT2, IFN-α, IFN-β) which are otherwise known to facilitate de novo T_H_1 formation [112]. A microarray-based multivariate analysis in the context of cedar pollinosis showed a differential apoptosis-related gene expression upregulation in CD4^+^T subset of the SLIT-treated group which suggests that induction of programmed cell death is a potential mechanism orchestrating the balance of all T cell subsets. However, it remains unclear which subpopulations were affected [120].

While the shifts in T_H_2/T_H_1 balance are often observed during or post treatment, it has also been found to have predictive potential at baseline. In the example of *Dermatophagoides pteronyssinus* (DP)-SCIT, patients who were responsive had a highly reactive pre-treatment state evidenced by low T_H_1 and high T_H_2 frequencies accompanied by higher concentrations of IL-5, IL-9 and IL-13. Contrastingly, non-responders showed a low reactivity profile at baseline defined by high T_H_1 and low T_H_2 proportions. The responsive group displayed a significant reduction in the proportion of T_H_2 cells after 12 months of SCIT while no shift was seen in non-responsive patients [100].

Besides affecting the overall T_H_2 subpopulation, a 1-year SLIT study demonstrated a reduction of particularly pathogenic HDM-reactive memory T_H_2 subsets defined as IL-5^+^IL-13^+^CD27^−^CD161^+^CD4^+^ and ST2^+^CD45RO^+^CD4^+^ the latter of which correlated with clinical benefit. Interestingly, these subpopulations in the non-responder group remained elevated making the subsets potential markers for treatment monitoring [121].

Critical T_FH_ role in the induction of IgE production constitutes them as a key subset to target by AIT. ICOS^+^ T_FH_ cells, IL-4^+^ T_FH_ cells, IL-21^+^ T_FH_ cells and dual IL-4^+^IL-21^+^ T_FH_ cells can be reduced by SCIT and SLIT [122•]. A recent study found that circulating Der p 1–specific IL-4^+^ T_FH_ cells can decrease as a result of AIT which correlated with the improvement of clinical symptoms. This shift was accompanied by a reduction of serum sIgE; however, no significant correlation between them suggests the contribution of alternative factors, such as regulatory counterparts T follicular regulatory (T_FR_) cells [37].

### Effect of AIT on Regulatory T Cells

Induction of regulatory T cell subsets is another key mechanism favoring tolerance. Both natural Tregs (nTregs) and induced Tregs (iTregs) modulate allergen-specific T_H_2 immunity with their respective mechanisms. nTregs is a thymus-derived subset defined by the expression of a transcription factor FOXP3 and surface marker CD25. The subpopulation is known to dampen the immune responses by direct cell-cell interaction in both healthy and allergic subjects. iTreg subpopulation arises in the periphery in response to the cytokine milieu and antigen stimulation and exerts their immunosuppressive abilities by secreting IL-10 and TGF-β accordingly comprising Tr1 and T_H_3 subsets [83]. IL-10, in particular, contributes to effector T cell anergy and is also crucial for the induction of allergen-specific IgG production [123]. Recently, the immunosuppressive capacity of newly identified IL-35 derived from the inducible regulatory T (iT_R_35) cells has been demonstrated following SLIT for grass pollen. In the treated group, the elevation of this subset was accompanied by IL-35-mediated dampening of cellular and humoral T_H_2 responses. Moreover, this cytokine exerted a suppressive capacity on IgE production in B cells [124••].

Current studies show that AIT can induce increases in circulating iTregs [101, 108], whereas FOXP3^+^CD25^+^ T cell role is rather attributed to increased local infiltration [102] or allergen-specific nTreg frequency and phenotypic shifts within the subpopulation [105]. Moreover, the immune response shift toward allergen-specific Tregs after 1 year of DP-SCIT is seen in patients who are responsive to the treatment [100].

A recent study proposed a regulatory role for IL-10-related transcription factor E4BP4, as its mRNA and IL-10 producing Tr1 were elevated in the blood of CP-SCIT-treated patients [103]. Special AT-rich sequence binding protein 1 (SATB1) has been identified as a transcriptional suppressor of FOXP3^+^ which in turn regulates Treg function. In an AIT cross-sectional study, both SCIT and SLIT were shown to increase DNA methylation levels at the SATB1 locus, subsequently resulting in reduced SATB1 mRNA expression, compared to untreated SAR group. Critically, SATB1 but not FOXP3 expression in Tregs correlated with the alleviation of clinical symptoms [125••]. Reduced sensitivity to apoptosis in FOXP3^+^ Tregs is another proposed AIT-induced mechanism of propelling tolerance, as seen in a murine asthma model [106].

Serine/threonine kinase CK2 is a constitutively active enzyme found to be crucial for the maintenance of a functional Treg phenotype in both humans and mice. Genetic ablation of the β-subunit of CK2 prompts the development of ILT3^+^ Treg subpopulation which is unable to modulate the airway T_H_2 response [126]. A study on Der p 1-specific FOXP3^+^ T cells after SCIT demonstrated a change of functional heterogeneity in the nTreg subset. The proportion of circulating activated allergen-specific FOXP3^+^Helios^+^ Tregs increased following a 30-week treatment and slightly declined at the 3-year mark, whereas the dysfunctional ILT3^+^ proportion was reduced. Importantly, these shifts correlated with improved allergic symptoms. The study also detected elevations of the Der p 1-specific IL-10^+^CD4^+^ T subset [105]. T_H_2 proliferation can directly be amplified by introducing antibodies against IL-10 and TGF-β into the supernatant of a co-culture system with effector and regulatory T cells isolated from patient PBMCs after 12 months of DP-SCIT. In the experiment, Tregs were also shown to have a greater capacity of suppressing IL-4 production in effector cells than those isolated pre-treatment [101]. A 3-year treatment with a grass sublingual tablet was shown to be key at inducing a lasting activated memory Treg phenotype defined as CD127^−^CD45RA^−^CD25^high^ in the circulation of 2/3 of the patients. Moreover, the response was associated with reduced eosinophil numbers as well as sIgE titers [98].

T_FR_ cells are regulatory counterparts of T_FH_ that express CXCR5, transcription factor FOXP3 and analogous surface molecules seen in Tregs which facilitate cell-cell contact-dependent immunosuppression. Interestingly, T_FR_ cells can migrate to germinal centers in a CXCR5-independent manner [127]. In AR patients, this subset is not only less abundant in the circulation, but also exhibits a reduced capacity to suppress T_FH_-mediated sIgE production. A 12-month HDM-SCIT demonstrated a significant restoration of the suppressive capacity on IgE production and an increase in circulating T_FR_ proportion. The latter correlated significantly with clinical improvement which potentially represents a biomarker for monitoring the treatment response [128]. A very recent study suggested that impaired chromatin accessibility and therefore immunosuppression-related gene transcription can contribute to the pathogenesis of SAR which importantly can be reversed by SCIT and SLIT [122•].

### B Cell-Related Tolerance

Upon AIT, allergen-specific IgE^+^ B cells switch isotype and start producing blocking antibodies with the same antigen specificity [84, 123]. This shift not only reduces the pathological IgE^+^ B cell subset, but also supplements antibodies that compete with the remaining sIgE for binding to the allergen.

IgE responses during AIT have been investigated in numerous studies. A study involving GP-SLIT demonstrated that IgE repertoire during the first year of treatment remains stable which suggests that there is no production of novel allergen-specific antibody clonotypes and therefore treatment-induced progression of the disease [46•]. Interestingly, in a DP-SCIT-responsive group, a significant sIgE elevation during initial months, which returned to baseline at year 1 of the treatment, was observed, whereas such shift was not seen in nonresponsive patients [100]. In a GRASS randomized clinical trial, SLIT was associated with a transient serum sIgE increase at year 1, while in SCIT group’s allergen-specific IgE concentration was not affect at the same time point and only decreased throughout years 2 and 3. Critically, this study demonstrated that 2 years of GP-SLIT is not sufficient to induce lasting clinical benefit at a 3-year follow up [129]. Alternatively, IgE responses can also be dampened by the downregulation of low affinity IgE receptor CD23 on switched memory B cells which was associated with positive clinical outcome after 12-month HDM-SCIT. This mechanism serves to impair T and B cell interaction which weakens antigen presentation and IgE synthesis [130•].

The ability to inhibit the effects of IgE resides mainly in IgG and IgA immunoglobulin fractions. AIT-induced IgG_4_ in particular is capable of binding to allergen epitopes otherwise recognized by IgE [84]. Thus, IgG dampens allergic inflammation by preventing further IgE production in B cells, mast cell and basophil degranulation and IgE-mediated antigen presentation by B and dendritic cells [85••, 89, 131]. AIT can induce blocking antibodies as early as 4 weeks into the treatment, while remaining elevation of this fraction at year 1 can predict patient responsiveness [100, 132]. After a 2-year increase in serum IgG_4_, the levels of this immunoglobulin stabilize during the third year and decrease post-therapy by 90%. However, the remaining circulating IgG exerts sufficient inhibitory capacity toward the activity of IgE which promotes lasting tolerance [90, 98]. Shamji et al. demonstrated that nasal secretions display a greater IgG-associated inhibitory activity than that of the serum in SCIT-treated patients. Remarkably, clinical efficacy of the treatment was shown to closely correlate with nasal IgG_4_ inhibitory capacity [85••]. The evidence of SLIT inducing B cell memory was observed in ryegrass-allergic patients as evidenced by elevation of allergen-specific IgG_2_ and IgG_4_ with corresponding IgG_2_^+^ and IgG_4_^+^ memory B cell frequencies which was associated with clinical improvement [133]. Timothy grass pollen SLIT treated children exhibited significantly higher levels of Phl p 1-specific salivary IgA and serum IgG_4_, along with lower SPT positivity after 3 years of treatment which proceeded throughout 2 years after treatment cessation [111].

Accumulating evidence highlights the importance of B regulatory cells (Bregs) for the induction of tolerance following AIT. A study by van de Veen et al. involving a 16-week bee venom-AIT elucidated key features and functions of this cell subset. Primarily defined by the expression of IL-10, these Breg cells were shown to have a CD25^high^CD71^high^CD73^low^ phenotype. During AIT, IL-10^+^ Breg cells become sole producers of allergen-specific IgG_4_, which demonstrates the dual capacity of this cell subset to induce tolerance both via allergen-specific T cell suppression and IgG-associated inhibitory processes [86]. Following this, numerous studies regarding seasonal and perennial inhaled allergens have reported AIT-induced increases in peripheral Breg frequencies and sIgG_4_. After a 2-year Der p1-SCIT, a significant elevation of serum allergen-specific and IgG_4_^+^ B cells was accompanied by the expansion of IL-10 and IL-1RA producing Bregs, which, taken together, correlated with a positive clinical outcome [87]. A 3-week therapy with linear rye-grass peptides was sufficient to alleviate seasonal symptoms in SAR patients. Aside from the induction of blocking IgG_4_ antibodies, the treatment also induced IL-10 production in Breg cells which was attributed to IL-35 secreted by the iT_R_35 subset [88•]. A cross-sectional controlled study demonstrated that SCIT-induced increases in IL-10^+^ Bregs were significantly higher during the natural pollen season than out of it. In a co-culture experiment, these CpG primed Bregs suppressed proliferation as well as IL-5 and IL-13 production in CD4^+^ T memory cells which was reversed by introducing anti-IL-10. Moreover, Breg frequencies in SCIT group correlated with serum sIgG_4_ and to a lesser extent—with nasal sIgG_4_ [85••]. Furthermore, a correlation between peripheral increases in grass pollen-specific IgG_4_ and IL-10^+^ Breg cells was observed after SCIT which potentially supports a previously observed notion that sIgG_4_ is produced by IL-10^+^ B cells [85••]. Induction of circulating IL-10^+^ Breg subpopulation has been suggested to have predictive value for anticipating treatment success, as increases of Breg/Th17 ratio at the initiation of the treatment significantly correlated with induction of tolerance at 3-year mark after GP-SCIT [134].

### Biomarkers of AIT

In order to ensure the maximum clinical benefit of AIT, there is a need to identify biomarkers to pre-select patients who are likely to become responders as well as biomarkers of desensitization, efficacy and tolerance. Currently, there are no confirmed biomarkers that can predict these parameters on an individual patient level [135]. Nevertheless, a recently published European Academy of Allergy and Clinical Immunology (EAACI) Position Paper reviewed current candidate biomarkers used to monitor the clinical efficacy of AIT in AR patients with or without asthma (Table 2) [142•].

**Table 2:** Six domains for monitoring the efficacy of allergen immunotherapy: antibodies, serum inhibitory activity for IgE, cytokines and chemokines, basophil activation, cellular, and in vivo biomarkers. *IgE-FAB*, IgE-facilitated antigen binding; *ELIFAB*, enzyme-linked immunosorbent-facilitated antigen binding; *DAO*, diamine oxidase

Domains	Biomarkers	References
Antibodies	IgE (tIgE, sIgE, sIgE/tIgE) sIgG4 sIgA	[20, 46•, 104, 136] [89, 102, 109, 137] [112]
Serum inhibitory activity for IgE	IgE-FAB ELIFAB	[89, 90, 138] [139]
Cytokines and chemokines	T_H_2-related: IL-4, IL-5, IL-9, IL-13 T_H_1-related: IFN-γ, CCL10, CCL11 Treg-related: IL-10, IL-35	[100, 108–110] [107, 110–112] [101, 125••]
Basophil activation	CD63/CD203c DAO	[95, 96••, 109, 140] [97]
Cellular biomarkers	Treg Breg DCreg	[100–106] [87–90] [27, 82]
In vivo	Allergen provocation tests Chamber studies	[94, 109, 112] [141]

### Mechanisms of AIT Determine Potential Biomarkers

Biomarkers for AIT monitoring consist of humoral, cellular and in vivo compartments. In serum, elevation of sIgE is considered to be the gold standard for both diagnosis of allergy and recruitment for AIT [19, 45, 98, 136]. Moreover, strong evidence supports the use of sIgE/tIgE ratio for predicting treatment efficacy [143]. Increases of serum sIgG_4_ have inconsistently been linked with the clinical outcome [100, 101], whereas the elevation of the same fraction and its inhibitory activity in the nasal mucosa were associated with ameliorated allergic response [85, 137]. To assess the overall serum inhibitory activity, attributed to IgG, IgA and IgD fractions, IgE-facilitated antigen binding (IgE-FAB) assay is conducted [85, 132, 138]. However, this assay is complex and may be limited to specialized facilities. An enzyme-linked immunosorbent-facilitated antigen binding (ELIFAB) is a more feasible assay for routine applications [139]. Reduction of inflammatory and increases in immunosuppressive chemokines and cytokines in serum are not seen consistently [100, 109, 111, 115], whilst nasally located mediators correlate with clinical benefit more frequently [93, 109]. In regard to cellular changes, AIT can alter cell function as well as subset frequencies and phenotypes. Reduction of basophil activation, determined by surface marker CD63 and CD203c expression, is inconsistently associated with lasting clinical improvement [94, 95, 100, 140]. Alternatively, reduction of basophil histamine release, determined by DAO assay, can correlate with AIT success [96••]. AIT-induced cellular changes, such as deviation toward DCreg [26, 81] and Treg phenotypes [98, 103, 105], are associated with induction of tolerance by skewing the immune response from T_H_2 to T_H_1. More recently, several studies have been able to associate clinical efficacy with the induction of IgG-producing memory B cells [133] or downregulation of their surface CD23 [130•]. Other recently arisen cellular candidates for monitoring AIT efficacy are pathogenic memory T_H_2 subsets [121], dysfunctional ILT3^+^ Tregs [105], Breg/Th17 ratio [134]. Notably, these findings remain to be replicated in future studies. Finally, in vivo biomarkers, such as allergen provocation tests and chamber studies, can be used to identify systemic or local sensitization to a relevant allergen [93, 100, 111, 141].

### Addressing the Unmet Needs and the Current Challenges in Biomarkers for AIT

Universal biomarkers for monitoring efficacy of AIT have yet to be identified and plenty remains to be understood. Nevertheless, with the recent advances in molecular and computational biology techniques, research limitations are as trivial as ever. The rise of machine learning-assisted unbiased clustering algorithms has revolutionized the interpretation of complex flow and mass cytometry data. As recent as they are, such computational tools have already elucidated mechanisms in fundamental immunology [144], cancer [145, 146], cell cycle [147], vaccination and HIV [148]. Importantly, unbiased analysis has substantially contributed to the understanding of immunological biomarkers in food allergy [149], and its usage for AR-related research in the future is more than likely. In the field of transcriptomics analysis, single-cell RNA sequencing (scRNA-seq) is the future of comprehensive and well-rounded understanding of biological processes. This analysis platform enables the assessment of gene expression in each individual cell which is superior to bulk analysis that often overlooks shifts in transcriptomic patterns by averaging the mRNA expression data [150]. In the field of allergy, scRNA-seq has already propelled knowledge of B cell class switching [151], B cell memory [46•], airway T cell metabolism [152], transcriptional differences between asthmatics with and without allergy [153], IgE responses [154], T cell clonotypes [155], and functional heterogeneity within allergen-specific T cell subset [156].

## Conclusions

AR is a serious health problem, affecting the quality of life of a vast proportion of the world’s population. Patients that do not respond to conventional anti-allergic medication can benefit from AIT which induces lasting clinical benefit post treatment cessation. Currently, there is a need for biomarkers to optimally select potentially responsive patients and monitoring the efficacy of the treatment. An in-depth understanding of both allergic inflammation and mechanisms of immunotherapy informs potential candidates for monitoring these parameters. Following nasal allergen challenge, EC-derived cytokines prime the development of pro-allergic DC2 and T_H_2 phenotypes. In the early phase, basophils and mast cells release their mediators upon surface IgE cross-linking by the allergen. These mediators recruit late phase effector T_H_2 cells which promote local eosinophilia. SCIT and SLIT for perennial and seasonal allergens dampen both innate and adaptive immune responses. Following AIT, reduced local infiltration of basophils, mast cells, eosinophils as well as their corresponding mediators is observed. Furthermore, induction of regulatory DC phenotype leads to the rise of Tregs which skew the immune response from T_H_2 to T_H_1 and give rise to the Breg subset. Breg and Treg-derived IL-10 prompts B cells isotype switch and subsequent IgG_4_ production. Informed by these mechanisms, current candidate biomarkers for monitoring AIT efficacy are classified into domains of sIgE, sIgG_4_, serum inhibitory activity, chemokines and cytokines, basophil activation, cellular changes, and in vivo biomarkers. However, there is no biomarker for monitoring clinical benefit on an individual patient level to date. Further studies need to be conducted to identify and confirm biomarkers, suitable for AIT efficacy monitoring and recognizing potential responders and non-responders.

## Abbreviations

AIT: Allergen immunotherapy
AR: Allergic rhinitis
Breg: B regulatory cell
CP: Cedar pollen
DAO: Diamine oxidase
DC: Dendritic cell
DP: *Dermatophagoides pteronyssinus* (en. house dust mite)
EC: Epithelial cell
ECP: Eosinophil cationic protein
FOXP3^+^: Forkhead box protein P3
GP: Grass pollen
HDM: House dust mite
IgE-FAB: IgE-facilitated antigen binding
ILC2: Group 2 innate lymphoid cell
NAC: Nasal allergen challenge
PAR: Perennial allergic rhinitis
PBMC: Peripheral blood mononuclear cell
PNIF: Peak nasal inspiratory flow
SAR: Seasonal allergic rhinitis
SATB1: Special AT-rich sequence binding protein 1
SCIT: Subcutaneous immunotherapy
SLIT: Sublingual immunotherapy
SPT: Skin prick test
T_FH_: T follicular helper cells
T_H_1: T helper type 1 cell
T_H_2: T helper type 2 cell
T_H_2A: Allergen-specific T_H_2 cells
TNSS: Total nasal symptom score
Treg: T regulatory cell
TSLP: Thymic stromal lymphopoietin
